# Diffusion basis spectrum imaging detects subclinical traumatic optic neuropathy in a closed-head impact mouse model of traumatic brain injury

**DOI:** 10.3389/fneur.2023.1269817

**Published:** 2023-12-13

**Authors:** Hsin-Chieh Yang, Raj Swaroop Lavadi, Andrew D. Sauerbeck, Michael Wallendorf, Terrance T. Kummer, Sheng-Kwei Song, Tsen-Hsuan Lin

**Affiliations:** ^1^Department of Radiology, Washington University School of Medicine, St. Louis, MO, United States; ^2^Department of Neurology, Washington University School of Medicine, St. Louis, MO, United States; ^3^Hope Center for Neurological Disorders, Washington University School of Medicine, St. Louis, MO, United States; ^4^Department of Biostatistics, Washington University School of Medicine, St. Louis, MO, United States; ^5^VA Medical Center, St. Louis, MO, United States

**Keywords:** traumatic optic neuropathy, traumatic brain injury, diffusion MRI, diffusion basis spectrum imaging, axonal loss, inflammation, modCHIMERA

## Abstract

**Introduction:**

Traumatic optic neuropathy (TON) is the optic nerve injury secondary to brain trauma leading to visual impairment and vision loss. Current clinical visual function assessments often fail to detect TON due to slow disease progression and clinically silent lesions resulting in potentially delayed or missed treatment in patients with traumatic brain injury (TBI).

**Methods:**

Diffusion basis spectrum imaging (DBSI) is a novel imaging modality that can potentially fill this diagnostic gap. Twenty-two, 16-week-old, male mice were equally divided into a sham or TBI (induced by moderate Closed-Head Impact Model of Engineered Rotational Acceleration device) group. Briefly, mice were anesthetized with isoflurane (5% for 2.5 min followed by 2.5% maintenance during injury induction), had a helmet placed over the head, and were placed in a holder prior to a 2.1-joule impact. Serial visual acuity (VA) assessments, using the Virtual Optometry System, and DBSI scans were performed in both groups of mice. Immunohistochemistry (IHC) and histological analysis of optic nerves was also performed after *in vivo* MRI.

**Results:**

VA of the TBI mice showed unilateral or bilateral impairment. DBSI of the optic nerves exhibited bilateral involvement. IHC results of the optic nerves revealed axonal loss, myelin injury, axonal injury, and increased cellularity in the optic nerves of the TBI mice. Increased DBSI axon volume, decreased DBSI λ_||_, and elevated DBSI restricted fraction correlated with decreased SMI-312, decreased SMI-31, and increased DAPI density, respectively, suggesting that DBSI can detect coexisting pathologies in the optic nerves of TBI mice.

**Conclusion:**

DBSI provides an imaging modality capable of detecting subclinical changes of indirect TON in TBI mice.

## Introduction

Traumatic brain injury (TBI) results in several, burdensome, ocular pathologies. A substantial proportion of patients with TBI experience visual problems resulting from optic neuropathy (1–5). Optic neuropathy in TBI can be direct or indirect. Direct traumatic optic neuropathy (TON) results from anatomical disruption of the optic nerve, whereas indirect TON originates from optic nerve stretch during a head injury. The latter can also occur due to secondary injury to the retinal ganglion cell axons (6, 7). Preclinical studies have showed that repeated mild TBI results in loss of the retinal nerve fiber layer and the inner plexiform layer (8–10). Changes in the pupillary diameter have also been noted following TBI, suggesting its neurovascular implications on the visual pathway in TBI (10). These changes may potentially root from a systemic and local dopaminergic disruption of the retina following TBI (10), and hemorrhage or edema (11–14). Both direct and indirect TON can contribute to optic nerve damage and a clear distinction is not always straightforward given that the optic nerve is vulnerable to multiple insults, such as the transmission of force or transient ischemia (12, 14).

TON is not commonly diagnosed due to slow disease progression, and undetected subclinical visual dysfunction. Missed diagnosis of indirect TON could lead to delayed or inappropriate treatment and subsequent reduction in visual function. Thus, an accurate and timely diagnosis of indirect TON is an unmet clinical need in treating patients with TBI. However, diagnosis of indirect TON is incomplete because standard visual functional assessments have limitations for appropriately reflecting optic nerve pathology (3, 15, 16). Tests such as optical coherence tomography (OCT) are also limited in detecting indirect TON as the accuracy of the retinal nerve fiber layer thickness can be confounded by inflammation-associated swelling in TBI (17). In addition, OCT cannot assess the posterior segment of the optic nerve, the most likely location of injury in indirect TON (12, 18).

To assess the posterior segment of the optic nerve, conventional magnetic resonance imaging (MRI) and diffusion tensor imaging (DTI) have seen increased application providing a comprehensive assessment of the visual system and related intracranial pathologies (19–21). However, both conventional MRI and DTI lack the needed specificity for the underlying pathologies in optic nerves (e.g., T2-hyperintense lesions can reflect demyelination, inflammation, or both, and changes in fractional anisotropy and apparent diffusion coefficient can result from axonal injury, demyelination, and/or inflammation) (20, 22–25).

To overcome the limitations of conventional MRI and DTI, diffusion basis spectrum imaging (DBSI) has been developed to detect, differentiate, and quantify coexisting pathologies (26), and to assess treatment efficacy (27, 28) in the optic nerve of a mouse model of multiple sclerosis. Aside from the application in optic nerve, DBSI has also demonstrated the ability to detect spinal cord axonal loss in patients with cervical spondylotic myelopathy (29, 30) and a mouse model of spinal cord injury (31) in the presence of tissue swelling. Thus, DBSI may hold the potential to specifically assess coexisting pathologies and reflect the corresponding severity of the underlying white-matter pathological components. In this study, DBSI was used to image the optic nerves of the murine moderate Closed-Head Impact Model of Engineered Rotational Acceleration (modCHIMERA) (32) to detect indirect TON and assess the progression of optic nerve pathology.

## Methods

All experimental procedures were approved by the Washington University Institutional Animal Care and Use Committee (IACUC) and performed according to the Public Health Service Policy on Humane Care and Use of Laboratory Animals (33).

### Experimental design

Twenty-two male C57BL/6 mice at age of 16 weeks old (The Jackson Laboratory, Bar Harbor, ME) were housed and maintained in the Washington University animal facility and subjected to a 12-h light/dark cycle. Eleven mice (five for *in vivo* and six for *ex vivo*) were injured as previously described using the modCHIMERA model (32). Briefly, mice were anesthetized with isoflurane (5% for 2.5 min followed by 2.5% maintenance during injury induction), had a helmet placed over the head, and were placed into a holder prior to a 2.1-joule impact (moderate—severe TBI) (32). The sham control group consisted of 11 (five for *in vivo* and six for *ex vivo*) mice undergoing the exact procedure without impact.

*In vivo cohort (n = 5 for each group)*: Visual acuity assessment and DBSI scans were performed at 1, 3, 7, and 30 days after impact or sham operation. At the end of the DBSI time course, the TBI and sham mice were deeply anesthetized and underwent perfusion-fixation via the left cardiac ventricle with phosphate buffered saline (PBS), followed by 4% paraformaldehyde (PFA). Brains were excised and stored in 4% PFA at 4°C for 24 h and then transferred to PBS for paraffin embedding processing. *Ex vivo cohort (n = 6 for each group)*: 3 days after impact or sham operation, mice were perfusion-fixed with 4% paraformaldehyde in PBS. After removing the skull, brain specimens were left in 4% paraformaldehyde for overnight at 4°C and then transferred to a 3-ml syringe filling with PBS for the *ex vivo* scan. After the *ex vivo* scan, the batch of the *ex vivo* cohort was processed with the same paraffin embedding processing protocol as the *in vivo* cohort.

### Visual acuity (for *in vivo* cohort only)

Mouse visual acuity (VA) was assessed using the Virtual Optometry System (Optometry, Cerebral Mechanics, Inc., Canada). Briefly, the virtual rotating columns were projected on the LCD monitors with different spatial frequencies in cycles/degree (c/d). The mouse head movement in response to the rotating virtual columns was noted. The VA was defined as the highest spatial frequency the mouse was able to respond to the virtual rotating columns. The spatial frequency was changed starting from 0.1 c/d with a step size of 0.05 c/d until the mouse stopped responding. When VA ≤ 0.25 c/d, the mouse was considered to develop impaired VA, as defined by previous studies (26, 27, 34, 35). VA was performed right before each *in vivo* MRI scan session.

### Magnetic resonance imaging

*In vivo* and *ex vivo* magnetic resonance imaging (MRI) experiments were performed on a 4.7-T Agilent DirectDrive™ small-animal MRI system (Agilent Technologies, Santa Clara, CA) equipped with a Magnex/Agilent HD imaging gradient coil (Magnex/Agilent, Oxford, United Kingdom). *In vivo DBSI protocol*: Mice were anesthetized with ~1% isoflurane/oxygen during scans. Breathing rate was monitored and body temperature was maintained at 37° C with a small animal physiological monitoring and control unit (SA Instruments, Stony Brook, NY). An actively decoupled 1.7-cm receive coil (36) was placed on top of the mouse head for MR signal reception. The animal holder assembly, containing the receive coil and monitoring accessories, was placed inside an 8-cm actively decoupled volume transmit coil, in a 12-cm cradle to be placed inside the magnet. 25-direction diffusion-weighted measurements were performed by a multi-echo spin-echo diffusion-weighting imaging sequence (37) with the following parameters: repetition time (TR) = 1.5 s; echo time (TE) = 35 ms; inter-echo delay = 20.7 ms; field of view (FOV) = 22.5 × 22.5 mm^2^; matrix size = 192 × 192 (zero-filled to 384 × 384), slice thickness = 1 mm, max; b value = 2,200 s/mm^2^, Δ = 18 ms; δ = 6 ms; total scan time = 124 min (26). *Ex vivo DBSI protocol*: The sample syringe was inserted into the solenoid surface coil to cover the whole brain. 99-direction diffusion-weighted measurements were performed by a multi-echo spin-echo diffusion-weighting imaging sequence (37) with the following parameters: TR = 3.0 s, TE = 31.5 ms, inter-echo delay = 23.4 ms, Δ = 18 ms, δ = 6 ms, maximal b-value = 3,000 s/mm^2^, with the same FOV = 15 × 15 mm^2^, slice thickness = 1 mm, and matrix size = 128 × 128 (zero-filled to 256 × 256). Total scan time was 10 h and 33 min.

### Imaging mouse optic nerve

The imaging site was at the pre-chiasmic segment of the optic nerve exhibiting symmetric left and right optic nerves in the axial view of the mouse brain. To maintain the consistency of imaging location, the multi-axial scout images were used to define mid-sagittal image slice (Supplementary Figure S1A). Mid-sagittal scout image with diffusion-weighting along the slice-selection direction was used to visualize corpus callosum (Supplementary Figure S1B, light blue arrow) and the optic nerve (Supplementary Figure S1B, green arrow). The reference axial image slice (Supplementary Figure S1B, red rectangle) was acquired perpendicular to the optic nerve, and the genu of corpus callosum was in the reference axial image slice (Supplementary Figure S1B, red rectangle). The cross-sectional optic nerves were at the final axial image with minimal confounding effects from cerebrospinal fluid (Supplementary Figure S1C).

### Diffusion MRI data analysis

Diffusion-weighted (DW) data were denoised (38) and converted into nifti format. The nifti-format DW data were analyzed with a lab-developed DBSI software package to perform DBSI multi-tensor analysis (20, 39). For optic nerve white matter tracts (coherent fiber bundles), the diffusion-weighted imaging data was modeled according to Equation 1:

(1) $$S_{k}=fe^{-\left|\vec{b_{k}}\right|\lambda_{⊥}}e^{-\left|\vec{b_{k}}\right|\left(\lambda_{\left|\right|}-\lambda_{⊥}\right)\cos^{2}\Phi_{k}}+\overset{b}{\underset{a}{\int }}f\left(D\right)e^{-\left|\vec{b_{k}}\right|D}dD\;\left(k=1,2,3,\ldots ,25\right)$$

The quantities $S_{k}$ and $\left|\vec{b_{k}}\right|$ are the signal and *b*-value of the *k^th^* diffusion gradient; $\Phi_{k}$ is the angle between the *k^th^* diffusion gradient and the principal direction of the anisotropic tensor; $\lambda_{\left|\right|}$ and $\lambda_{⊥}$ are the axial and radial diffusivities of the anisotropic tensor, respectively; f is the signal intensity fraction for the anisotropic tensor; *a* and *b* are the low and high diffusivity limits for the isotropic diffusion spectrum, reflecting cellularity and edema, respectively, f(D) . DBSI-derived f represents optic nerve white-matter tract density in an image voxel. DBSI-derived λ_||_ and λ_⊥_ reflect residual axon and myelin integrity, respectively: ↓ λ_||_ ≈ axonal injury and ↑ λ_⊥_ ≈ myelin damage (20, 39–41). As dictated by previous experimental findings, the restricted isotropic diffusion fraction reflecting cellularity in mouse optic nerves is derived by the summation of f(D) at 0 ≤ apparent diffusion coefficient (ADC) ≤ 0.6 μm^2^/ms. The summation of the remaining f(D) at 0.6 < ADC ≤ 2 (for *ex vivo*) and 0.6 < ADC ≤ 3 (for *in vivo*) μm^2^/ms represents non-restricted isotropic diffusion, which putatively denotes vasogenic edema and cerebrospinal fluid (20, 39–41).

Regions of interest (ROI) were manually drawn in the center of each optic nerve on the diffusion-weighted image, which corresponded to the diffusion gradient direction perpendicular to optic nerves, to minimize partial volume effects. ROIs were then transferred to the parametric maps to calculate the mean for each of the DBSI metrics.

### ROI for DBSI-derived axon volume

Separate ROIs encompassing the whole optic nerve for axon volume calculation were drawn on the diffusion weighted images (DWI) orthogonal to the optic nerve, which were larger than the ROIs for DBSI metrics. The fiber fraction was measured of this larger ROI. DBSI-derived axon volume was calculated from the optic nerve volume (the entire ROIs on DWIs) multiplying by corresponding DBSI fiber fraction of this larger ROI.

### Immunohistochemistry

Optic nerve pairs with chiasm were dissected (Supplementary Figure S2A) and embedded in 2% agar first (42). The agar block was embedded with liquid paraffin and the optic nerve was placed at the bottom of the embedding mold. The paraffin blocks were then sectioned from the optic chiasm at 20-μm thickness. Once passing the region of the optic chiasm, the sectioning thickness was reduced to 5-μm for 40 slices. The sectioning region reached the MRI imaging region (Supplementary Figure S2A, yellow rectangle), and the cross-sectional left and right optic nerves were shown as two circles (Supplementary Figure S2B, red arrows). The paraffin sectioning slices were deparaffinized and rehydrated for immunohistochemistry (IHC) analysis. Three nerves were damaged during the extraction process, and the other 41 nerves were used for paraffin embedding process. Sections were blocked with 5% normal goat serum and 1% bovine serum albumin in PBS for 30 min at room temperature to prevent non-specific binding. Slides were then incubated overnight at 4°C with primary antibody and then 1 h at room temperature with the appropriate secondary antibody. Primary antibodies used were anti-total neurofilament (SMI-312, staining both injured and intact axons, BioLegend, 1:300), anti-phosphorylated neurofilament (SMI-31, reflecting intact axons, BioLegend, 1:300), and anti-myelin basic protein (MBP, assessing myelin sheaths, Sigma, 1:300). Secondary antibodies were goat anti-mouse or goat anti-rabbit (Invitrogen, 1:240), which were conjugated to either Alexa 488 for SMI-31, SMI-312, or MBP. Slides were mounted with Vectashield Mounting Medium for DAPI (4′, 6-dianidino-2-phenylindole, detecting cell nuclei, Vector Laboratory Inc., Burlingame, CA) and coverslipped. Images were acquired on a Nikon Eclipse 80i fluorescence microscope with MetaMorph software (Universal Imaging Corporation, Sunnyvale, CA) at 100× magnifications.

### Histological data analysis

The whole field of SMI-31, MBP, and DAPI staining images at 100× magnification was captured with the same fluorescent light intensity and exposure time. All captured images were converted to 8-bit grayscale and analyzed using threshold, edge enhancement, analyze particles, and gray level watershed segmentation functions of ImageJ.^1^

### Statistical analysis

The correlations of histology and VA data with DBSI measurements were analyzed by a mixed random effect regression that accounted within mouse correlation between eye measurements. MRI measurements were taken from all eyes of sham and TBI mice. A mixed two-way ANOVA was performed between sham and TBI groups for comparing DBSI, DTI, and histology data. The correlation of histology data and DBSI measurements was analyzed by using Pearson Correlation Coefficients.

## Results

### Inconsistency between visual acuity and *in vivo* DBSI assessments

**Figure 1:**
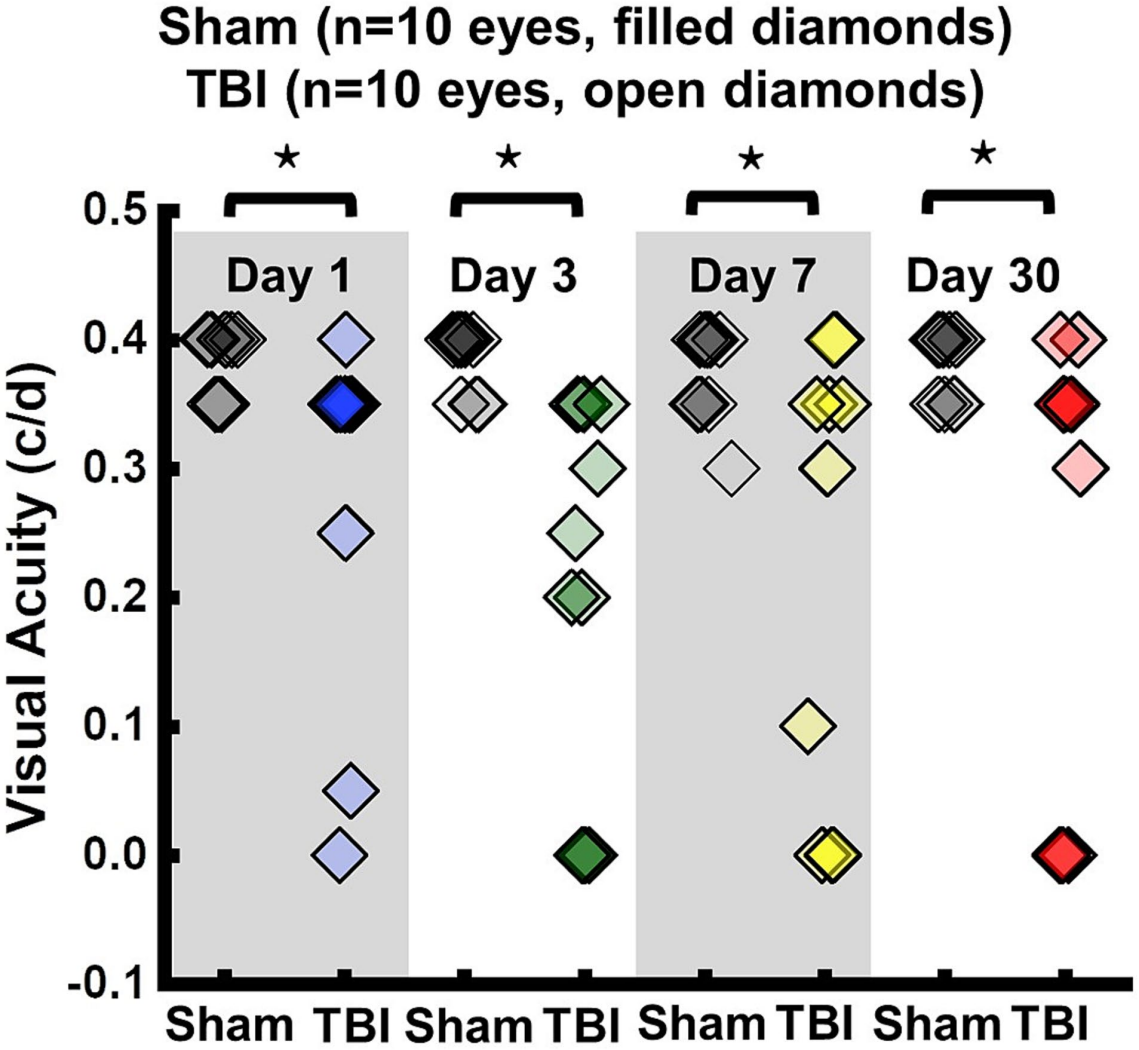
Longitudinal visual acuity (VA) of sham (filled diamonds) and TBI (open diamonds) eyes 1, 3, 7, and 30 days after TBI. VA was impaired in 3 out of 10 eyes, 9 out of 10 eyes, 4 out of 10 eyes, and 3 out of 10 eyes, at 1, 3, 7, and 30 days after TBI, respectively. ⋆ indicates *p* < 0.05.

VA of TBI mice was unilaterally or bilaterally affected (Figure 1). At 1 day after TBI, VA was impaired in 3 out of 10 eyes and progressed to 9 out of 10 impaired eyes at 3 days after TBI. VA improved to impairment in 4 out of 10 and 3 out of 10 eyes at 7 and 30 days after TBI, respectively (Figure 1). DBSI metrics of optic nerves, however, were bilaterally affected with decreased DBSI-λ_||_, elevated DBSI-λ_⊥_, increased DBSI restricted (putative inflammatory cellularity) fraction, and decreased fiber fraction at 1, 3, 7, and 30 days after TBI (Table 1).

**Table 1:** Quantitative longitudinal DBSI/DTI results of sham and TBI optic nerves.

*In vivo*		**Day 1**	**Day 3**	**Day 7**	**Day 30**
DBSI non-restricted fraction	Sham	0.03 ± 0.043	0.05 ± 0.043	0.06 ± 0.035	0.06 ± 0.054
	TBI	0.03 ± 0.029	0.05 ± 0.025	0.07 ± 0.054	0.10 ± 0.048
DBSI restricted fraction	Sham	0.03 ± 0.014	0.03 ± 0.018	0.04 ± 0.036	0.05 ± 0.014
	TBI	0.12 ± 0.050^⋆^	0.11 ± 0.046^⋆^	0.12 ± 0.056^⋆^	0.18 ± 0.069^⋆^
DBSI-derived Axon Volume (mm^3^)	Sham	0.11 ± 0.009	0.10 ± 0.006	0.11 ± 0.009	0.11 ± 0.007
	TBI	0.10 ± 0.009	0.11 ± 0.009^⋆^	0.09 ± 0.014^⋆^	0.08 ± 0.022^⋆^
DBSI λ_∥_ (μm^2^/ms)	Sham	1.89 ± 0.128	1.89 ± 0.092	1.84 ± 0.088	1.84 ± 0.210
	TBI	1.47 ± 0.178^⋆^	1.59 ± 0.163^⋆^	1.55 ± 0.161^⋆^	1.61 ± 0.211^⋆^
DBSI λ_⊥_ (μm^2^/ms)	Sham	0.15 ± 0.038	0.17 ± 0.056	0.15 ± 0.039	0.14 ± 0.024
	TBI	0.18 ± 0.029	0.22 ± 0.041^⋆^	0.21 ± 0.044^⋆^	0.23 ± 0.053^⋆^
DTI λ_∥_ (μm^2^/ms)	Sham	1.72 ± 0.124	1.75 ± 0.058	1.68 ± 0.066	1.62 ± 0.183
	TBI	1.19 ± 0.229^⋆^	1.21 ± 0.220^⋆^	1.20 ± 0.199^⋆^	1.07 ± 0.184^⋆^
DTI λ_⊥_ (μm^2^/ms)	Sham	0.17 ± 0.046	0.20 ± 0.051	0.19 ± 0.040	0.20 ± 0.058
	TBI	0.20 ± 0.025	0.26 ± 0.039^⋆^	0.28 ± 0.046^⋆^	0.34 ± 0.060^⋆^
DTI FA	Sham	0.88 ± 0.040	0.86 ± 0.039	0.87 ± 0.032	0.85 ± 0.062
	TBI	0.81 ± 0.042^⋆^	0.75± 0.059^⋆^	0.71 ± 0.089^⋆^	0.61 ± 0.0117^⋆^
DTI MD (μm^2^/ms)	Sham	0.23 ± 0.012	0.24 ± 0.008	0.23 ± 0.009	0.22 ± 0.015
	TBI	0.18 ± 0.027^⋆^	0.19± 0.024^⋆^	0.20 ± 0.019^⋆^	0.19 ± 0.013^⋆^

*indicates *p* < 0.05.

**Figure 2:**
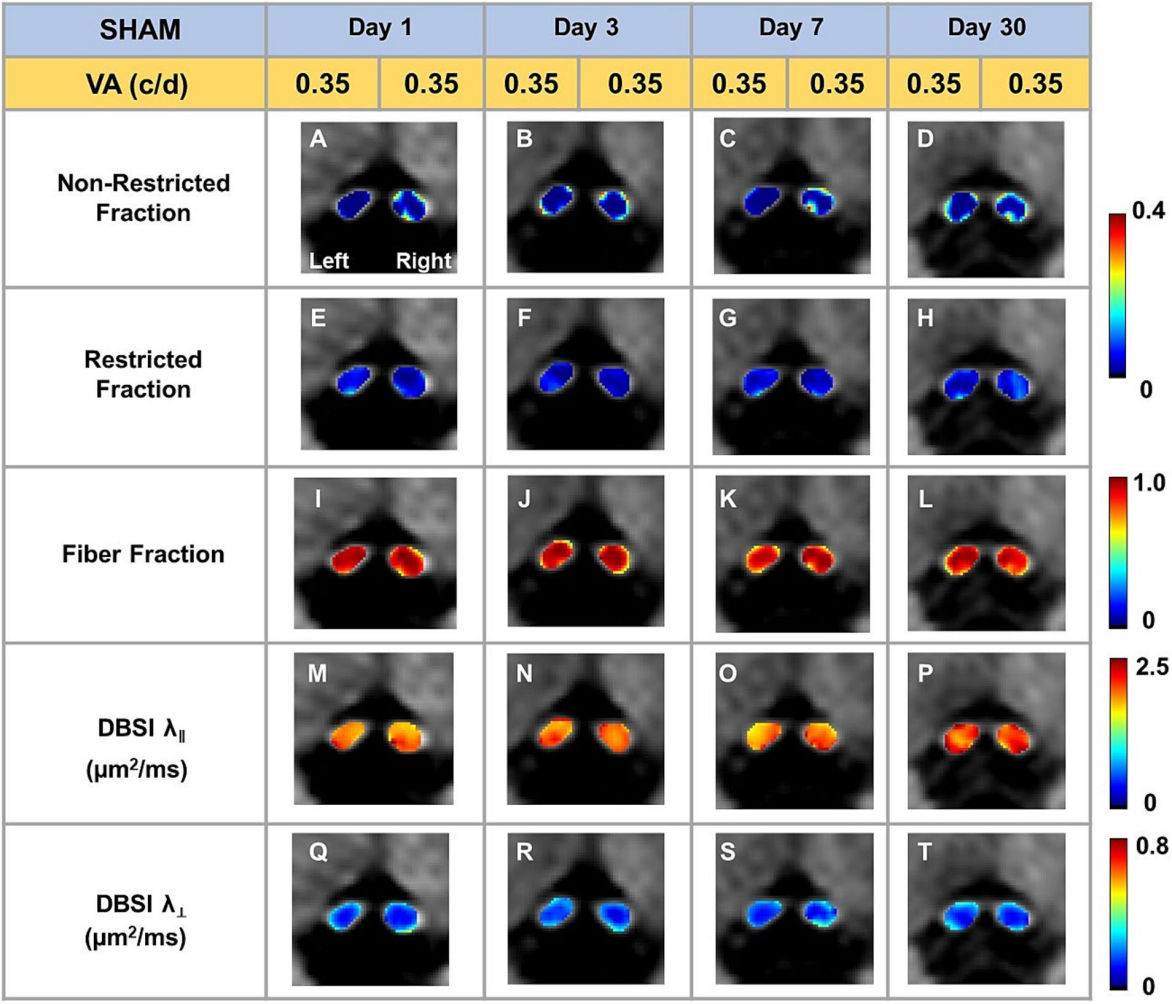
Representative DBSI metrics were overlaid on gray-scale diffusion-weighted images of sham optic nerves. DBSI non-restricted fraction **(A–D)**, restricted fraction **(E–H)**, Fiber fraction **(I–L)**, DBSI axial diffusivity (λ_‖_) **(M–P)**, and DBSI radial diffusivity (λ_┴_) **(Q–T)** were consistent at, 1, 3, 7, and 30 days after TBI. The corresponding VA was at the normal score all the time.

**Figure 3:**
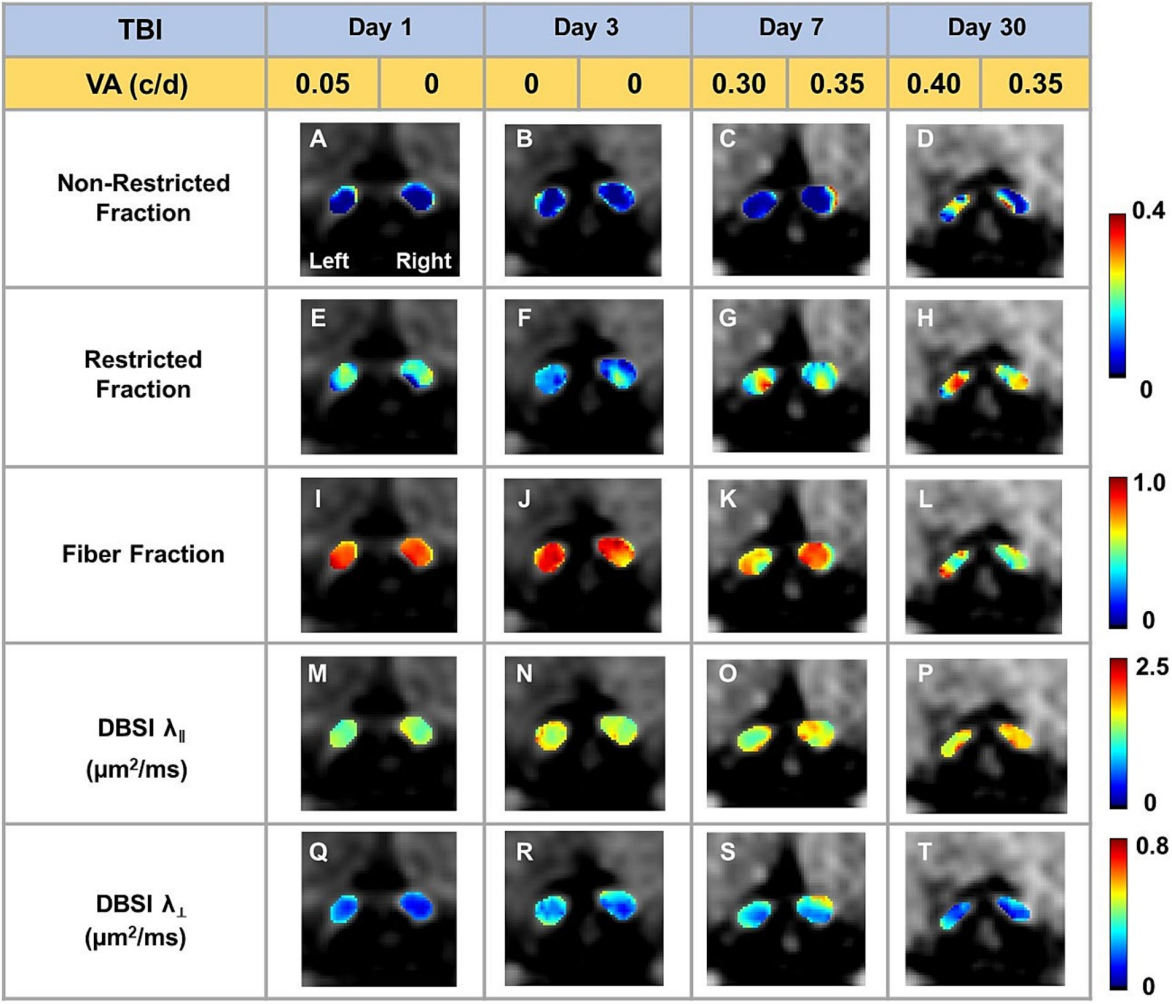
Representative DBSI metrics were overlaid on gray-scale diffusion-weighted images of TBI optic nerves. Compared to sham optic nerves in Figure 2, elevated DBSI restricted fraction **(E–H)**, reduced fiber fraction **(I–L)**, reduced DBSI axial diffusivity (λ_‖_) **(M–P)**, and increased DBSI radial diffusivity (λ_┴_) **(Q–T)** were shown at 1, 3, 7, and 30 days after TBI. The corresponding VA was not consistent with DBSI metrics.

Representative DBSI metrics and VA from one sham mouse at 1, 3, 7, and 30 days after sham operation demonstrated that DBSI metrics describe normal nerve structure, consistent with normal VA (Figure 2). Comparing to sham optic nerves, the increased DBSI restricted fraction (Figures 3E–H), decreased fiber fraction (Figures 3I–L), and decreased DBSI-λ_||_ in the representative TBI mouse optic nerves at 1, 3, 7, and 30 days after injury, suggesting increased cellularity, reduced axonal density, and axonal injury. The increased DBSI-λ_⊥_ was shown at 3, 7, and 30 days after injury (Figures 3R–T), indicating myelin injury in the TBI optic nerves. In addition, the increased non-restricted fraction and optic nerve shrinkage were shown at 30 days after injury (Figures 3D,H,L,P,T), reflecting vasogenic edema and atrophy. Moreover, the volume of the optic nerve was 0.117 (left) and 0.151 (right) mm^3^ vs. 0.148 (left) and 0.172 (right) mm^3^ at 1 and 3 days after injury, respectively, while the fiber fraction remained unchanged (Figures 3I,J), suggesting cytotoxic edema of the optic nerves at the acute stage after TBI. The corresponding VA in TBI was inconsistent with optic nerve pathologies (Figure 3).

### *In vivo* DBSI metrics reflected coexisting pathologies in the TON optic nerve

**Figure 4:**
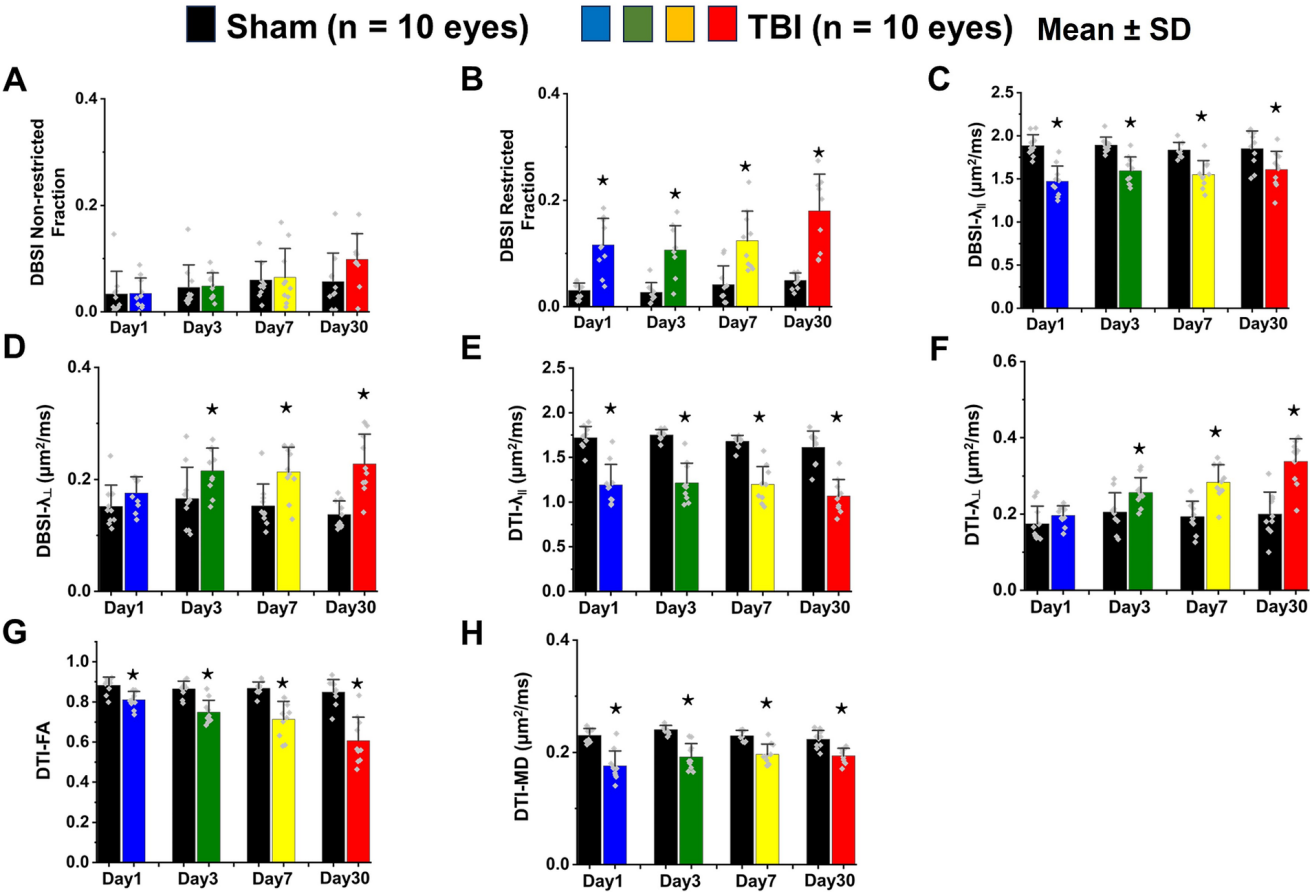
Bar graphs of DBSI **(A–D)** and DTI **(E–H)** metrics. ^⋆^ indicates *p* < 0.05.

DBSI metrics could reflect coexisting pathologies in the optic nerve of TBI mice during the observation period up to 30 days after TBI. Comparing to sham mice, the DBSI non-restricted fraction (putative biomarker of vasogenic edema) was not significantly different from 1, 3, 7, and 30 days after TBI (Figure 4A; Table 1). DBSI restricted fraction (Figure 4B; Table 1) elevated, compared to sham mice, by 300% (0.12 ± 0.050 vs. 0.03 ± 0.014, TBI vs. sham, *p* < 0.05), 267% (0.11 ± 0.046 vs. 0.03 ± 0.018, TBI vs. sham, *p* < 0.05), 200% (0.12 ± 0.056 vs. 0.04 ± 0.036, TBI vs. sham, *p* < 0.05), and 260% (0.18 ± 0.069 vs. 0.05 ± 0.014, TBI vs. sham, *p* < 0.05) at 1, 3, 7, and 30 days, respectively, after TBI. DBSI-λ_||_ (Figure 4C; Table 1) decreased by 22% (1.89 ± 0.128 vs. 1.47 ± 0.178 μm^2^/ms, Sham vs. TBI, *p* < 0.05), 16% (1.89 ± 0.092 vs. 1.59 ± 0.638 μm^2^/ms, Sham vs. TBI, *p* < 0.05), 15% (1.84 ± 0.088 vs. 1.55 ± 0.161 μm^2^/ms, Sham vs. TBI, *p* < 0.05), and 13% (1.84 ± 0.210 vs. 1.61 ± 0.211 μm^2^/ms, Sham vs. TBI, *p* < 0.05) at 1, 3, 7, and 30 days, respectively, after TBI. DBSI-λ_⊥_ (Figure 4D; Table 1) increased by 16% (0.15 ± 0.038 vs. 0.18 ± 0.029 μm^2^/ms, Sham vs. TBI, *p* = 0.13), 30% (0.17 ± 0.056 vs. 0.22 ± 0.041 μm^2^/ms, Sham vs. TBI, *p* < 0.05), 29% (0.15 ± 0.039 vs. 0.21 ± 0.044 μm^2^/ms, Sham vs. TBI, *p* < 0.05), and 34% (0.14 ± 0.024 vs. 0.23 ± 0.053 μm^2^/ms, Sham vs. TBI, *p* < 0.05) at 1, 3, 7, and 30 days, respectively, after TBI.

### *In vivo* DTI metrics were confounded by coexisting pathologies in the TON optic nerve

In contrast to DBSI metrics, DTI-λ_||_ (Figure 4E; Table 1) and DTI-λ_⊥_ (Figure 4F; Table 1) showed an exaggerated decrease and increase, respectively, suggesting DTI-derived axonal injury and myelin injury were confounded by inflammation and axonal loss. Comparing to the sham mouse optic nerve, reduced DTI fractional anisotropy (FA; Figure 4G; Table 1) and DTI mean diffusivity (MD, Figure 4H; Table 1) reflected the combined effects of axonal injury, inflammation, and axonal loss.

### *In vivo* DBSI detected axonal loss in the TON optic nerve

**Figure 5:**
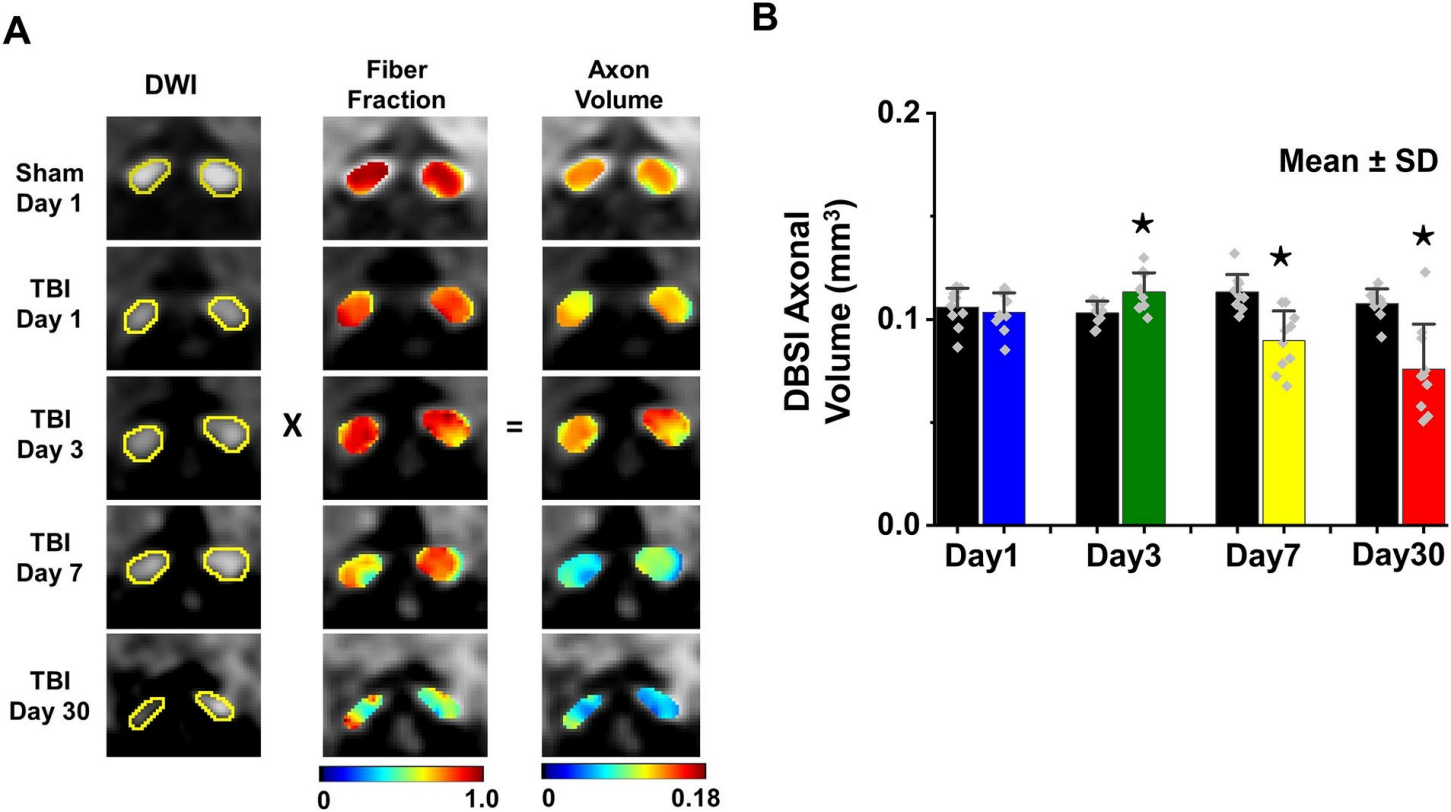
DBSI-axonal volume (unit in mm^3^) was derived by multiplying nerve volume (unit in mm^3^) with fiber fraction **(A)**. DBSI-derived axonal volume of sham and TBI optic nerves from 1, 3, 7, and 30 **(B)**. ^⋆^ indicates *p* < 0.05.

DBSI-derived fiber fraction may reflect apparent fiber (i.e., axon) density that is confounded by dilution effects from other pathological components. DBSI-derived fiber fraction decreased by 7% (0.81 ± 0.060 vs. 0.76 ± 0.038, Sham vs. TBI, *p* < 0.05), 1% (0.77 ± 0.031 vs. 0.76 ± 0.024, Sham vs. TBI, *p* = 0.29), 6% (0.76 ± 0.044 vs. 0.72 ± 0.065, Sham vs. TBI, *p* = 0.10), and 20% (0.81 ± 0.082 vs. 0.61 ± 0.107, Sham vs. TBI, *p* < 0.05) at 1, 3, 7, and 30 days, respectively, after TBI. To quantify the extent of axons, a quantitative metric was developed to remove the dilution effects resulting from vasogenic edema and other complications. The metric was developed by multiplying DBSI anisotropic fiber fraction with optic nerve volume (assessed by conventional or diffusion-weighted MRI) resulting in a metric reflecting axon content with, i.e., *axon (fiber) volume* (Figure 5A). The nerve volume assessed using diffusion-weighted images with DW direction perpendicular to the fiber tract changed 4% (0.13 ± 0.011 vs. 0.14 ± 0.011 mm3, Sham vs. TBI, *p* = 0.03), 12% (0.13 ± 0.006 vs. 0.15 ± 0.011 mm3, Sham vs. TBI, *p* < 0.05), −17% (0.15 ± 0.011 vs. 0.12 ± 0.017 mm3, Sham vs. TBI, *p* < 0.05), and − 15% (0.14 ± 0.018 vs. 0.12 ± 0.016 mm3, Sham vs. TBI, *p* < 0.05) at 1, 3, 7, and 30 days, respectively, after TBI (Table 1). DBSI-derived axon volume decreased 2% (0.11 ± 0.009 vs. 0.10 ± 0.009 mm^3^, Sham vs. TBI, *p* = 0.58), −10% (0.10 ± 0.006 vs. 0.11 ± 0.009 mm^3^, Sham vs. TBI, *p* < 0.05), 21% (0.11 ± 0.009 vs. 0.09 ± 0.014 mm^3^, Sham vs. TBI, p < 0.05), and 30% (0.11 ± 0.007 vs. 0.08 ± 0.022 mm^3^, Sham vs. TBI, p < 0.05) at 1, 3, 7, and 30 days, respectively, after TBI (Figure 5B; Table 1).

### Optic nerve axonal injury at 3 days after TBI (*ex vivo* cohort)

DBSI non-restricted fraction was not significant differences between the sham and TBI group (Table 2). DBSI restricted fraction (Table 2) was elevated, compared to sham mice, by 47% (0.19 ± 0.055 vs. 0.10 ± 0.055, TBI vs. sham, *p* < 0.05). DBSI-λ_||_ (Table 2) decreased by 28% (0.74 ± 0.090 vs. 0.53 ± 0.070 μm^2^/ms, Sham vs. TBI, *p* < 0.05). DBSI-λ_⊥_ (Table 2) increased by 50% (0.06 ± 0.026 vs. 0.09 ± 0.017 μm^2^/ms, Sham vs. TBI, *p* < 0.05). DBSI fiber fraction decreased by 9% (0.68 ± 0.050 vs. 0.62 ± 0.096 μm^2^/ms, Sham vs. TBI, *p* = 0.06). The nerve volume assessed using diffusion-weighted images with DW direction perpendicular to the fiber tract changed by 10% (0.10 ± 0.012 vs. 0.09 ± 0.018 mm^3^, Sham vs. TBI, *p* = 0.13). DBSI-derived axon volume (Table 2) exhibited a 14% decrease (0.07 ± 0.009 vs. 0.06 ± 0.015 mm^3^, Sham vs. TBI, *p* < 0.05). Comparing DBSI metrics, DTI-λ_||_ (Table 2) and DTI-λ_⊥_ (Table 2) showed an exaggerated decrease and increase, respectively. Comparing to the sham mouse optic nerve, reduced DTI-FA (Table 2) and DTI-MD (Table 2) were observed.

**Table 2:** Quantitative DBSI/DTI results of sham and TBI optic nerves at Day 3 (*ex vivo* cohort).

*Ex vivo*		Day 3
DBSI non-restricted fraction	Sham	0.06 ± 0.026
	TBI	0.07 ± 0.058
DBSI restricted fraction	Sham	0.10 ± 0.042
	TBI	0.19 ± 0.055^⋆^
DBSI-derived axon volume (mm^3^)	Sham	0.07 ± 0.009
	TBI	0.06 ± 0.015^⋆^
DBSI λ_∥_ (μm^2^/ms)	Sham	0.74 ± 0.090
	TBI	0.53 ± 0.070^⋆^
DBSI λ_⊥_ (μm^2^/ms)	Sham	0.06 ± 0.026
	TBI	0.09 ± 0.017^⋆^
DTI λ_∥_ (μm^2^/ms)	Sham	0.61 ± 0.090
	TBI	0.42 ± 0.075^⋆^
DTI λ_⊥_ (μm^2^/ms)	Sham	0.08 ± 0.071
	TBI	0.12 ± 0.036^⋆^
DTI FA	Sham	0.85 ± 0.071
	TBI	0.68 ± 0.102^⋆^
DTI MD (μm^2^/ms)	Sham	0.09 ± 0.015
	TBI	0.07 ± 0.013^⋆^

*indicates *p* < 0.05.

**Figure 6:**
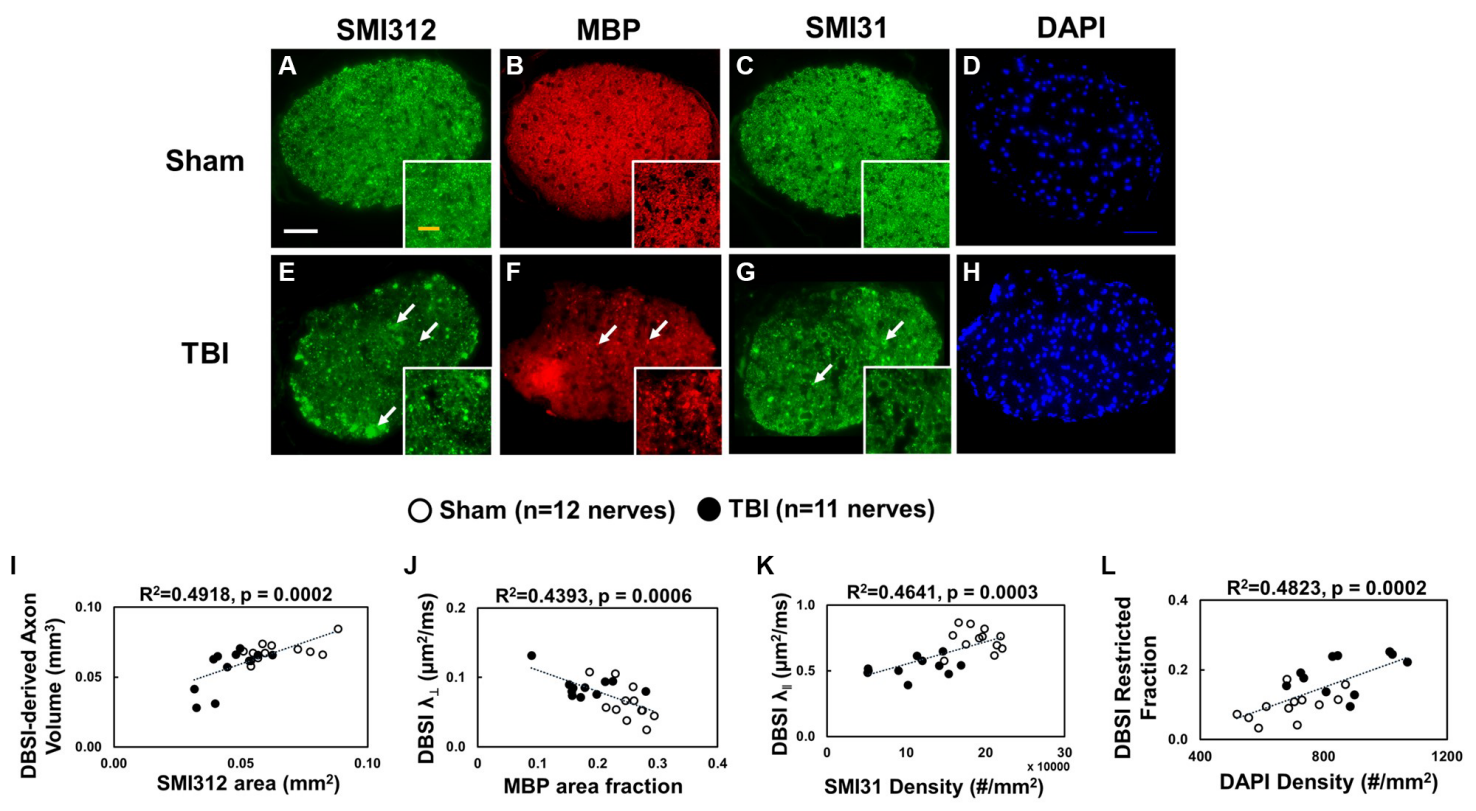
Representative 100× IHC from one sham **(A–D)** and one TBI **(E–H)** optic nerve (tissues were processed after *ex vivo* DBSI, 3 days after impact). The correlation of SMI-312 area, MBP area fraction, SMI-31 and DAPI density with DBSI-derived axon volume **(I)**, DBSI λ_⊥_ **(J)**, DBSI λ_||_ **(K)**, and DBSI restricted isotropic fraction **(L)** suggested that DBSI was able to reflect the severity of axonal loss, myelination, axonal integrity, and inflammatory-increased cellularity, respectively. White scale bar: 50 μm; Yellow scale bar: 25 μm.

Representative IHC results revealed axonal loss (reduced SMI-312 area; Figures 6A,E), demyelination (reduced MBP area fraction; Figures 6B,F), severe axonal swelling (Figures 6E–G, white arrows), axonal injury (reduced SMI-31 density; Figures 6B,C,G) and increased cellularity (increased DAPI density; Figures 6D,H) in the optic nerves of the TBI mice. The correlations of DBSI-derived axon volume (Figure 6I, r^2^ = 0.4918, *p* = 0.0002), DBSI λ_⊥_ (Figure 6J, r^2^ = 0.4393, *p* = 0.0006), DBSI λ_||_ (Figure 6K, r^2^ = 0.4641, *p* = 0.0003), and DBSI restricted fraction (Figure 6L, r^2^ = 0.4823, *p* = 0.0002) were consistent with SMI-312 area, MBP area fraction, SMI-31 density, and DAPI density, suggesting that DBSI biomarkers reflect coexisting pathologies of the optic nerves in the TBI mice. The reduced SMI-312 area (0.064 ± 0.013 vs. 0.045 ± 0.001 mm^2^, Sham vs. TBI, *p* < 0.05; Table 3), and MBP area fraction (0.25 ± 0.03 vs. 0.18 ± 0.05, Sham vs. TBI, *p* < 0.05; Table 3), SMI-31 density (189,969 ± 24,492 vs. 108,139 ± 42,917 #/mm^2^, Sham vs. TBI, *p* < 0.05; Table 3) in the TBI group suggested axonal loss, demyelination, and axonal injury in their optic nerves. The increased DAPI density (690 ± 109 vs. 864 ± 129 #/mm^2^, Sham vs. TBI, *p* < 0.05; Table 3) in the TBI group suggested area increased cellularity in the TBI optic nerves.

**Table 3:** Quantitative IHC results of sham and TBI optic nerves at Day 3 (*ex vivo* cohort).

*Ex vivo*	SMI312 (mm^2^)	MBP fraction	SMI31 (#/mm^2^)	DAPI (#/mm^2^)
Sham	0.064 ± 0.013	0.25 ± 0.03	189,969 ± 24,492	690 ± 109
TBI	0.045 ± 0.001^⋆^	0.18 ± 0.05^⋆^	108,139 ± 42,917^⋆^	864 ± 129^⋆^

*indicates *p* < 0.05.

### IHC of optic nerves at the end of longitudinal MRI

**Figure 7:**
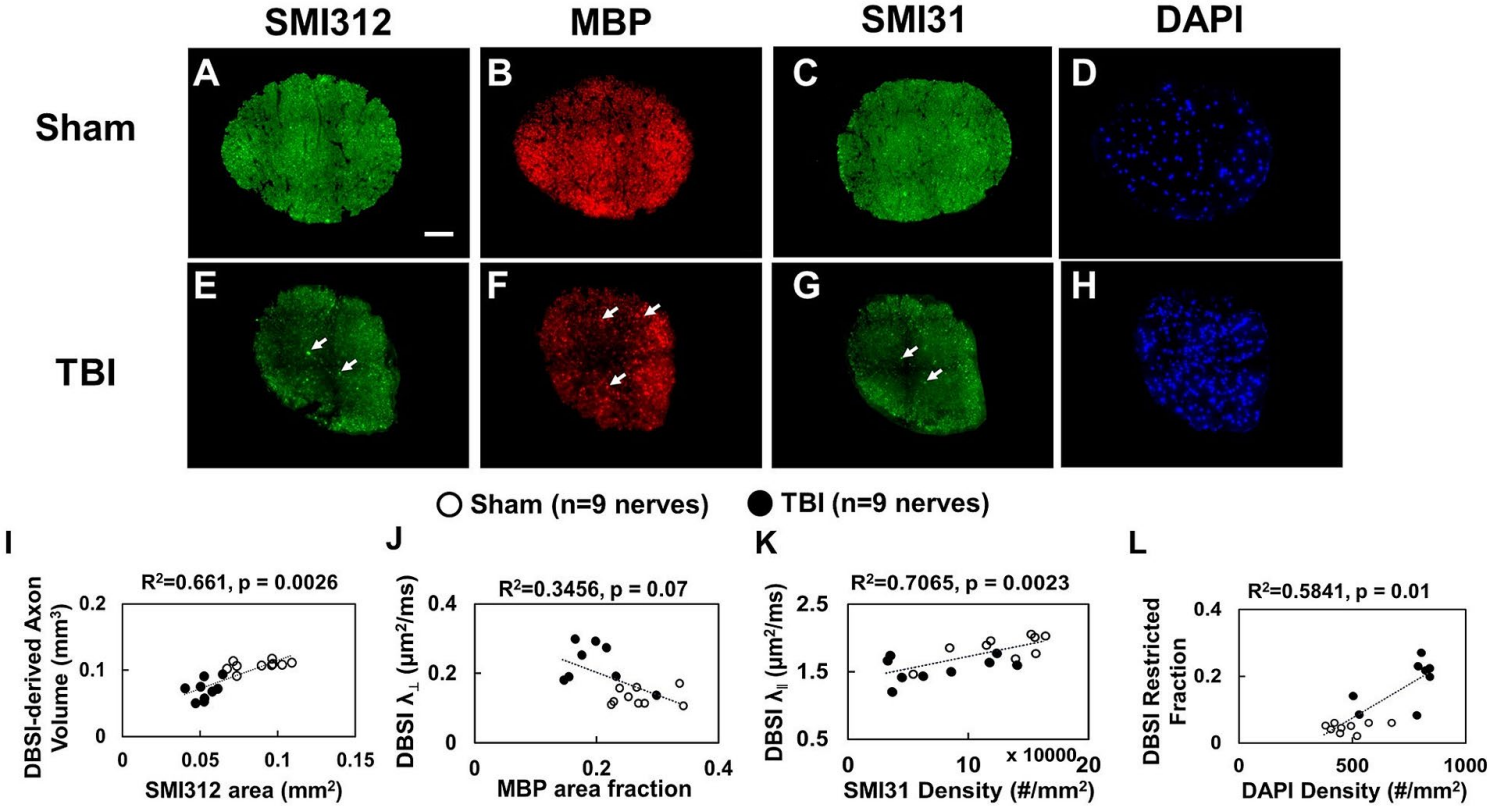
Representative 100× IHC from one sham **(A–D)** and one TBI **(E–H)** optic nerve (tissues were saved right after *in vivo* DBSI, 30 days after impact). The correlation of SMI-312 area, MBP area fraction, SMI-31 and DAPI density with DBSI-derived axon volume **(I)**, DBSI λ_⊥_ **(J)**, DBSI λ_||_ **(K)**, and DBSI restricted isotropic fraction **(L)** suggested that DBSI was able to reflect the severity of axonal loss, myelination, axonal integrity, and inflammatory-increased cellularity, respectively. White scale bar: 50 μm.

Representative IHC of optic nerves revealed axonal loss (reduced SMI-312 intensity; Figures 7A,E), myelin injury (reduced MBP area fraction; Figures 7B,F), axonal injury (reduced SMI-31 density; Figures 7C,G), and increased cellularity (increased DAPI density; Figures 7D,H) in the optic nerves of TBI mice. The correlations of DBSI-derived axon volume (Figure 7I, r^2^ = 0.661, *p* = 0.0026), DBSI λ_⊥_ (Figure 7J, r^2^ = 0.3456, *p* = 0.07), DBSI λ_||_ (Figure 7K, r^2^ = 0.7065, *p* = 0.0023), and DBSI restricted fraction (Figure 7L, r^2^ = 0.5841, *p* = 0.01) was consistent with SMI-312 area, MBP intensity, SMI-31 density, and DAPI density, suggesting that DBSI reflected coexisting pathologies in the optic nerves of TBI mice. The reduced SMI-312 area (0.088 ± 0.015 vs. 0.053 ± 0.007 mm^2^, Sham vs. TBI, *p* < 0.05; Table 4), MBP area fraction (0.27 ± 0.04 vs. 0.20 ± 0.05, Sham vs. TBI, *p* < 0.05; Table 4), SMI-31 density (125,197 ± 37,281 vs. 74,336 ± 42,548 #/mm^2^, Sham vs. TBI, *p* < 0.05; Table 4) in the TBI group suggested axonal loss, demyelination, and axonal injury in the TBI optic nerves. The increased DAPI density (479 ± 92 vs. 745 ± 133 #/mm^2^, Sham vs. TBI, *p* < 0.05; Table 4) in the TBI group suggested increased cellularity in the TBI optic nerves.

**Table 4:** Quantitative IHC results of sham and TBI optic nerves at Day 30 (*in vivo* cohort).

*In vivo*	SMI312 (mm^2^)	MBP fraction	SMI31 (#/mm^2^)	DAPI (#/mm^2^)
Sham	0.088 ± 0.015	0.27 ± 0.04	125,197 ± 37,281	479 ± 92
TBI	0.053 ± 0.007^⋆^	0.20 ± 0.05^⋆^	74,336 ± 42,548^⋆^	745 ± 133^⋆^

*indicates *p* < 0.05.

## Discussion

In the current study, longitudinal DBSI and VA assessments were performed to investigate optic nerve pathology at 1, 3, 7, and 30 days after TBI. The results suggested that DBSI detected axonal injury, myelin damage, axonal loss, and inflammatory cellularity in the optic nerves of mice with TBI. The axonal loss identified in this study indicates that axonal volume is a valid marker of axonal extent. This study shows that optic nerve changes induced by TBI can be reproduced in the modCHIMERA TBI mice.

To evaluate the indirect TON, VA examinations were performed. Previously, it was identified that VA was sensitive to identify the onset of acute optic neuritis in myelin oligodendrocyte glycoprotein (MOG)-induced experimental autoimmune encephalomyelitis (EAE) mice (26, 28, 34, 35). In the current study, VA of TBI mice was unilaterally or bilaterally affected (Figure 1), but coexisting pathologies were detected in both optic nerves (Figure 3; Table 1). The current data indicated that VA does not reflect the optic nerve pathology of indirect TON in modCHIMERA mice. VA impairment in these mice may be confounded by psychophysical deficits in the modCHIMERA model as suggested by previously reported social and motor deficits (32), although the motor deficits are short lived.

Optic neuritis is one of the prominent and early pathologies of EAE mice (43). In acute EAE mice, change in VA was consistent with optic nerve pathology before chronic neurological deficits interfered with the optomotor reflex assessment (26, 34, 35). In indirect TON, a compensatory mechanism might play a role in the normalization of VA in the chronic stage (44). Therefore, VA may not be sensitive enough for visual function evaluation in indirect TON of TBI mouse models. For a more accurate assessment of visual function in indirect TON, behavioral independent assessments would improve the analyses.

Optic nerve pathologies in indirect TON include cytotoxic edema, vasogenic edema, and inflammation with activated microglia and macrophages (45, 46), followed by Wallerian degeneration (47, 48). Decreased apparent diffusion coefficient has been shown to associate with optic nerve injury and to be used as a surrogate marker of sight loss in unconscious patients (49). The decreased axial and mean diffusivity, believed to reflect damage of the axolemma and axonal swelling, has been proposed to serve as a biomarker of axonal damage and as a predictor of initial visual acuity and potential visual recovery in patients with TON (6). The current findings in TBI mice are consistent with TON patients exhibiting decreased DTI and DBSI derived axial diffusivity although the decreased axial diffusivity was seen in optic nerves of normal and impaired visual acuity in TBI mice. The results suggest the presence of subclinical optic nerve pathologies that may escape timely detection and treatment in TON.

A previous study has identified that DBSI derived axon volume detected axonal loss in the presence of white matter tract swelling (26). Another marker of axonal swelling is decreased radial diffusivity, as a result of axonal crowding (6). The clinical correlation of nerve swelling is questionable and seems unpredictable. Stahl et al. presented a 21-year-old male patient with right-sided visual loss after sustaining a penetrating orbital trauma, with minimal ocular trauma, from a metal rod (49). The MRI showed a unilateral reduction of the subarachnoid space of the optic nerve on the affected side, with a normal optic nerve diameter. The patient had permanent visual loss. Becker et al. presented a case whereby a patient suffered from a skull base fracture and had axonal injury of the optic nerve (21). DWI showed widening of the left optic nerve at the level of the orbital apex. This patient also had permanent visual loss.

DBSI quantitatively assesses axonal volume that is capable of quantifying the cytotoxic edema induced axonal swelling without being confounded by extra-fiber swelling resulting from inflammatory cell infiltration and vasogenic edema (assessed by non-restricted diffusion). This has been consistent with our previous study showing DBSI quantified axonal loss in the presence of nerve swelling in acute spinal cord injury (31). Thus, DBSI complements the conventional MRI measured cross-sectional areas of optic nerves and spinal cords by quantitatively distinguishing and assessing intra- and extra-fiber swelling.

In this study, it was identified that significantly increased nerve volume can be attributed to increased DBSI restricted fraction (extra-axonal swelling), increased DBSI axon volume (intra-axonal swelling), unchanged DBSI fiber fraction (axonal density), and unchanged DBSI non-restricted fraction (extra-axonal swelling, i.e., vasogenic edema) at 3 days after TBI. For the *in vivo* cohort, the acute nerve swelling can be partially attributed to the axonal swelling (i.e., cytotoxic edema) as reflected by the increased DBSI-derived axonal volume at 3 days after TBI. To further investigate the cytotoxic-edema impact of DBSI-derived axonal volume at 3 days after TBI, the *ex vivo* cohort was used to validate the correlation between DBSI and histology (Figure 7; Table 2) since histological fixation typically dehydrates and shrinks the processed tissue, masking vasogenic edema induced tissue swelling (50). The *ex vivo* brain samples underwent 24-h 4% PFA fixation, then transferred to the PBS solution prior to the DBSI scan. The *ex vivo* nerve volume at 3 days after TBI was examined for detecting cytotoxic edema with minimized contribution of vasogenic edema. DBSI-derived axonal volume at 3 days after TBI detected axonal loss consistent with the histological results (Figure 7).

Although DTI metrics may not reflect the true axonal and myelin pathologies due to confounds from coexisting pathologies such as inflammatory cell infiltration and vasogenic edema as previously reported (22–25). Current longitudinal diffusion-weighted MRI examination of optic nerves in mice revealed the same trend of both DTI and DBSI derived axial (λ_||_, decreased comparing with that of sham) and radial (λ_⊥_, increased comparing with that of sham) diffusivity in TBI mice, suggesting axonal and myelin injury. This is consistent with a previously reported retrospective investigation on the diffusion-weighted MRI signal intensity of the optic nerve in 29 patients with TON (51), where a hyperintense signal was seen in injured eyes. The current study revealed an increased DTI λ_⊥_ and DBSI λ_⊥_ from days 1 to 30 after TBI, suggesting myelin injury. This is consistent with the reduced MBP area fraction at 3 and 30 days after TBI. A similar extent of myelin injury was identified by Qiu et al., who examined NF-1 and MBP in a repetitive mild TBI mouse model 7-months after injury and found significantly reduced expression of both proteins when compared with sham animals (52). Khan et al. reported similar observations in their repetitive TBI mice model (53).

Although the current study did not examine the retinal pathology or a specific mechanism of TON along the entire optic nerve tract, the localized findings are encouraging and validate the utility of DBSI to detect and distinguish optic nerve injury types and components. The results demonstrated the presence of TON in modCHIMERA-induced TBI mice. Future studies may consider exploring the intersegmental changes of the optic nerve in a TBI animal model (6, 51) and variations in injury patterns using DBSI and resting state functional imaging (54, 55). The results from the current study should be interpreted under the context of the limitations of modCHIMERA-induced TBI in mice (32). This study may serve as a catalyst to encourage bench-to-bedside efforts to detect TON using a novel imaging modality and potentially reduce the burden of TON in patients with TBI.

## Conclusion

DBSI and DTI suggested subclinical injury of indirect TON in TBI mice. However, DTI-derived metrics are confounded by coexisting pathologies, lacking the needed accuracy or specificity in detecting optic nerve injuries. DBSI is capable of detecting and distinguishing coexisting optic nerve pathologies. DBSI is potentially more effective for detecting the clinically silent optic nerve pathologies in indirect TON. Thus, DBSI may serve as an outcome measure of TON.

## Data availability statement

The raw data supporting the conclusions of this article will be made available by the authors, without undue reservation.

## Ethics statement

The animal study was approved by Washington University Institutional Animal Care and Use Committee. The study was conducted in accordance with the local legislation and institutional requirements.

## Author contributions

H-CY: Data curation, Investigation, Validation, Visualization, Writing – original draft, Writing – review & editing. RL: Data curation, Validation, Writing – original draft, Writing – review & editing. AS: Data curation, Methodology, Writing – review & editing. MW: Data curation, Methodology, Writing – review & editing. TK: Writing – review & editing. S-KS: Conceptualization, Validation, Writing – original draft, Writing – review & editing, Funding acquisition. T-HL: Conceptualization, Data curation, Investigation, Methodology, Project administration, Resources, Software, Supervision, Validation, Visualization, Writing – original draft, Writing – review & editing.

## Footnotes

^1^ http://bigwww.epfl.ch/sage/soft/watershed/
