# Supplementary figures and images for: Diffusion basis spectrum imaging detects subclinical traumatic optic neuropathy in a closed-head impact mouse model of traumatic brain injury
Source: Front Neurol. 2023 Dec 13;14:1269817. doi: 10.3389/fneur.2023.1269817 (PMC10752006; doi:10.3389/fneur.2023.1269817)

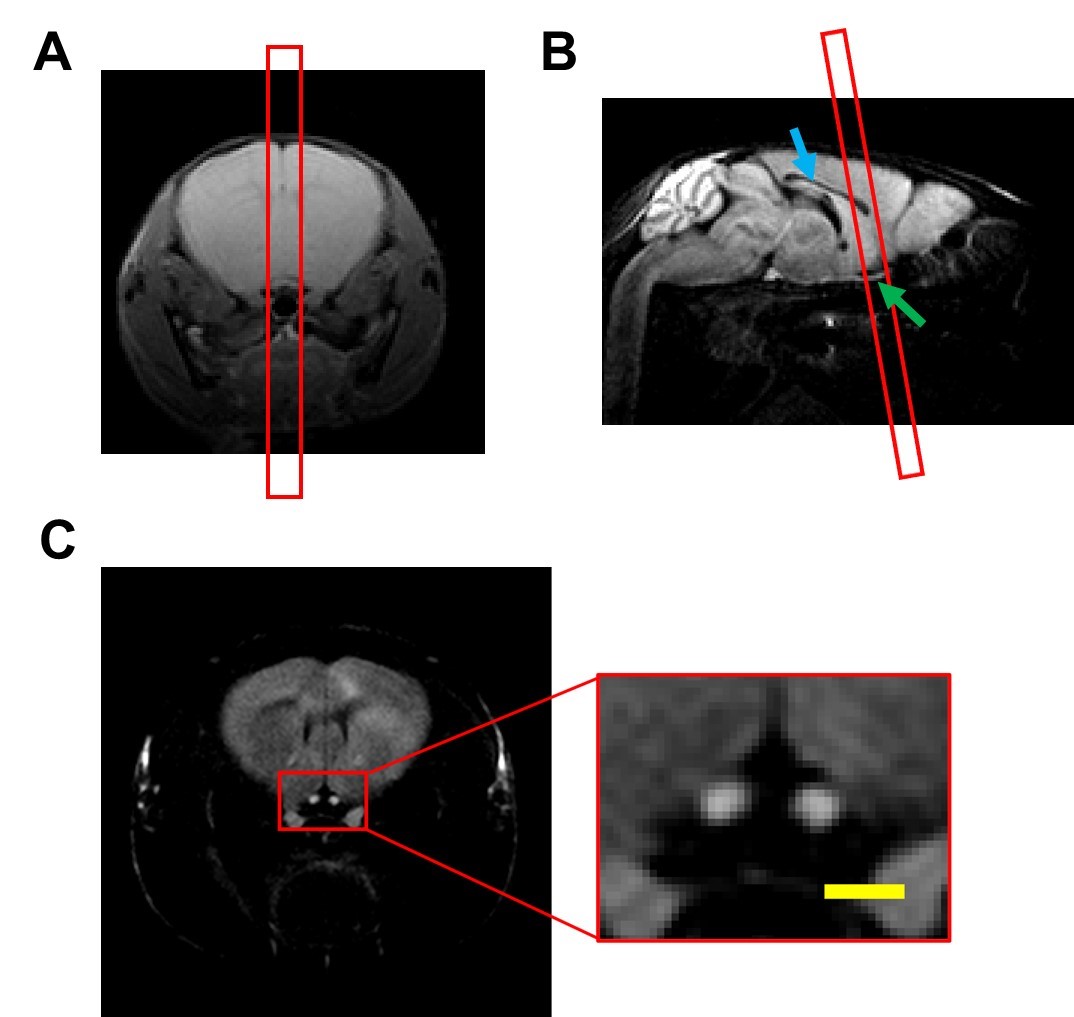

Supplement: Supplementary file 1 [file Image_1.jpg]

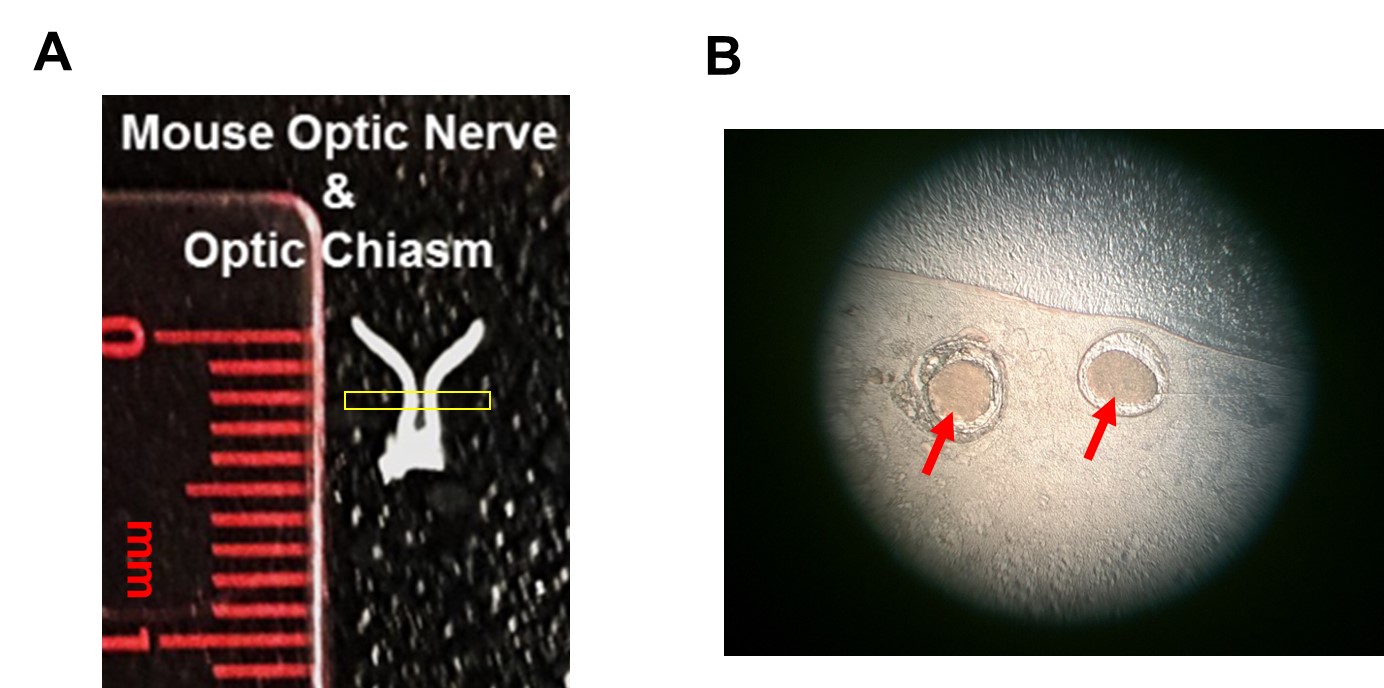

Supplement: Supplementary file 2 [file Image_2.jpg]
